# Intranasal Allopregnanolone Confers Rapid Seizure Protection: Evidence for Direct Nose-to-Brain Delivery

**DOI:** 10.1007/s13311-020-00985-5

**Published:** 2021-01-06

**Authors:** Dorota Zolkowska, Chun-Yi Wu, Michael A. Rogawski

**Affiliations:** 1 Department of Neurology, School of Medicine, University of California, Davis, Sacramento, CA 95817 USA; 2 Bioanalysis and Pharmacokinetics Core Facility, UC Davis Medical Center, Sacramento, CA 95817 USA; 3 Department of Pharmacology, School of Medicine, University of California, Davis, Sacramento, CA 95817 USA

**Keywords:** Allopregnanolone, Neuroactive steroid, Intranasal delivery, Nose-to-brain, Seizure, Pharmacokinetics

## Abstract

Allopregnanolone, a positive modulator of GABA_A_ receptors with antiseizure activity, has potential in the treatment of seizure emergencies. Instillation of allopregnanolone in 40% sulfobutylether-β-cyclodextrin into the nose in mice rapidly elevated the seizure threshold in the timed intravenous pentylenetetrazol (ED_50_, 5.6 mg/kg), picrotoxin (ED_50_, 5.9 mg/kg), and bicuculline seizure tests. The effect peaked at 15 min, decayed over 1 h, and was still evident in some experiments at 6 h. Intranasal allopregnanolone also delayed the onset of seizures in the maximal PTZ test. At an allopregnanolone dose (16 mg/kg) that conferred comparable effects on seizure threshold as the benzodiazepines midazolam and diazepam (both at doses of 1 mg/kg), allopregnanolone caused minimal sedation or motor toxicity in the horizontal screen test whereas both benzodiazepines produced marked behavioral impairment. In addition, intranasal allopregnanolone failed to cause loss-of-righting reflex in most animals, but when the same dose was administered intramuscularly, all animals became impaired. Intranasal allopregnanolone (10 mg/kg) caused a rapid increase in brain allopregnanolone with a *T*_max_ of ~5 min after initiation of the intranasal delivery. High levels of allopregnanolone were recovered in the olfactory bulb (*C*_max_, 16,000 ng/mg) whereas much lower levels (*C*_max_, 670 ng/mg) were present in the remainder of the brain. We conclude that the unique ability of intranasal allopregnanolone to protect against seizures without inducing behavioral adverse effects is due in part to direct nose-to-brain delivery, with preferential transport to brain regions relevant to seizures. Benzodiazepines are commonly administered intranasally for acute seizure therapy, including for the treatment of acute repetitive seizures, but are not transported from nose-to-brain. Intranasal allopregnanolone acts with greater speed, has less propensity for adverse effects, and has the ability to overcome benzodiazepine refractoriness. This is the first study demonstrating rapid functional central nervous system activity of a nose-to-brain-delivered steroid. Intranasal delivery circumvents the poor oral bioavailability of allopregnanolone providing a route of administration permitting its evaluation as a treatment for diverse neuropsychiatric indications.

**Supplementary Information:**

The online version contains supplementary material available at 10.1007/s13311-020-00985-5.

## Introduction

The neuroactive steroid allopregnanolone (brexanolone) is an endogenous metabolite of progesterone that acts as a positive allosteric modulator of GABA_A_ receptors [47]. An intravenous formulation of allopregnanolone is approved as a treatment for postpartum depression [50]. Allopregnanolone may also be useful in the treatment of other diverse neurological and psychiatric conditions, including seizures and epilepsy. The potential use in the acute treatment of seizure emergencies is particularly compelling as there is evidence in animal models that allopregnanolone effectively terminates benzodiazepine refractory seizures [48]. Following systemic administration, allopregnanolone readily enters the brain [63]. However, it has poor (~2%) oral bioavailability [40]. In clinical use, the steroid is therefore administered as an intravenous formulation, restricting broad applicability of the treatment. An additional drawback to intravenous allopregnanolone is the risk for excessive sedation [46, 53].

In the present study, we sought to assess whether allopregnanolone can be administered via the nose as a means to overcoming its poor oral bioavailability. Intranasal delivery provides a non-invasive route of administration that bypasses gastrointestinal and hepatic first-pass metabolism [9, 20]. Additionally, the intranasal route may offer onset of action comparable in speed to intravenous delivery [60]. There is substantial variability in the bioavailability of organic molecules when administered via the nose. However, certain steroids, including the sex steroids progesterone [12, 16, 56], estradiol [56, 57] and testosterone [5, 13, 62] are well absorbed intranasally and are concentrated in the brain. There is evidence that these steroids are not only absorbed into the capillaries of the nasal mucosa and then transported from the venous circulation across the blood–brain barrier into the brain but that a portion of the dose is transported directly from nose-to-brain, bypassing the bloodstream. Thus, following deposition into the nose, the relative concentrations in certain brain regions including the olfactory bulbs, which are adjacent to the nasal cavity, were shown to be higher than those in other brain regions [5, 16]. An objective of the present study was to determine if a portion of nasally administered allopregnanolone similarly passes directly into the brain.

Benzodiazepines are the current standard-of-care for the treatment of acute seizure emergencies. Intranasal delivery provides certain advantages to other routes of administration [11, 30]. Currently, intranasal formulations of diazepam and midazolam are approved for the treatment of acute repetitive seizures. In the present study, we observed that intranasal allopregnanolone has antiseizure activity in the mouse, demonstrating that intranasal delivery is a feasible route of administration. However, we unexpectedly noted that intranasal allopregnanolone failed to induce transitory neurological impairment whereas doses of diazepam and midazolam that produced a similar magnitude elevation of seizure threshold were strongly sedating. Measurements of allopregnanolone levels in the brain provided evidence for direct nose-to-brain delivery suggesting that preferential exposure of brain regions relevant to seizures accounts for the ability of intranasal allopregnanolone to impact seizures with minimal acute toxicity.

## Methods

### Animals

NIH Swiss male mice (22–32 g) from Charles River were kept in a vivarium under controlled environmental conditions (22–26 °C; 40–50% humidity) with an artificial 12-h light/dark cycle. Food and water were provided ad libitum. Experiments were performed during the light phase of the light/dark cycle after a minimum 30-min period of acclimation to the experimental room. The animal facilities were fully accredited by the Association for Assessment and Accreditation of Laboratory Animal Care. All studies were performed under protocols approved by the Animal Care and Use Committee of the University of California, Davis in strict compliance with the *Guide for the Care and Use of Laboratory Animals, Eighth Edition* (The National Academies Press, 2011).

### Test Substances and Drug Administration

Allopregnanolone (custom synthesized, > 99% pure) was dissolved at a concentration of 16 mg/ml in an aqueous solution containing 0.9% NaCl and 40% sulfobutylether-β-cyclodextrin (Captisol®; Ligand Pharmaceuticals, San Diego, CA). The allopregnanolone solution was instilled in the nose using a series of small volume drops over the course of 3.5 min to reach a total dose of 2.5–16 mg/kg. Intranasal drug administration was carried out in awake mice as described by Hanson et al. [23]. In brief, the mouse was scruffed and turned onto the back, so the mouse’s back was laying in the palm of the experimenter’s hand. The mouse’s chin and neck were positioned in line and parallel to the floor. Treatments were administered into the external nares as 2–3 μl drops with a Hamilton microliter syringe equipped with the blunt 30 G needle. After each administration, the mouse was held in the supine position for at least 15 s. The animal was then allowed to rest in its home cage for 30–60 s.

Pharmaceutical-grade midazolam and diazepam were purchased from Hospira (Lake Forest, IL); the solution concentrations were 1 mg/ml and 5 mg/ml, respectively. Midazolam and diazepam were administered intranasally at a dose of 1 mg/kg using the same procedure as allopregnanolone. Vehicle solutions were prepared using the excipients as in the commercial products. The vehicle for midazolam solution contained 0.8% NaCl, 0.01% EDTA disodium salt, and 1% benzyl alcohol adjusted to pH 3.3 to 3.5. The vehicle for diazepam solution contained 40% propylene glycol, 10% ethyl alcohol, 5% sodium benzoate and benzoic acid as buffers, and 1.5% benzyl alcohol. Pentylenetetrazol (PTZ), picrotoxin, and (+)-bicuculline were purchased from MilliporeSigma (previously Sigma-Aldrich). PTZ was used as a 10 mg/ml solution in 0.9% NaCl and bicuculline was used as a 0.1 mg/ml solution in slightly acidic water. Picrotoxin was prepared as a 1 mg/ml solution in 0.9% NaCl.

### Timed Intravenous Seizure Threshold Test in Mice

The thresholds for various behavioral seizure stages induced by the PTZ, picrotoxin, and bicuculline were determined by infusing the convulsant drugs via a 27-gauge, 0.75-in. “butterfly” needle inserted into the lateral tail vein. PTZ (10 mg/ml), picrotoxin (1 mg/ml) and bicuculline (0.1 mg/ml) were infused IV at a constant rate of 0.5 ml/min using a 5-ml syringe (BD Biosciences, Franklin Lakes, NJ) mounted on an infusion pump (Model ‘11’ Plus Syringe Pump; Harvard Apparatus, Holliston, MA). The syringe was connected to the needle by polyethylene tubing. The infusion was stopped at 3 min or at the onset of tonic extension, whichever occurred first. The time to occurrence (“infusion duration”) to the following endpoints from the start of the convulsant infusion were determined: (1) the first myoclonic body twitch; (2) the onset of generalized clonus; and (3) the onset of tonic extension. Infusion duration values represent the time from the start of the convulsant infusion to the onset of the endpoint. The threshold value (mg/kg) for each endpoint was determined according to the following formula: (infusion duration [s] × infusion rate [ml/min] × convulsant concentration [10 mg/ml, 1 mg/ml or 0.1 mg/ml] × 1000)/(60 s × body weight of mouse [g]).

### Maximal PTZ Seizure Test

Mice were injected intraperitoneally with PTZ (80 mg/kg) and were observed for a 30-min period. The times to occurrence of the 3 seizure signs as in the timed intravenous PTZ seizure test were recorded. Allopregnanolone (10 mg/kg) or vehicle was administered intranasally 5 min or 10 min prior to the PTZ injection.

### Horizontal Screen Test

At various times after intranasal administration, mice were placed on a horizontally oriented grid (consisting of parallel 1.5-mm diameter rods situated 1 cm apart) and the grid was slowly inverted over 2–3 s and held approximately 30–40 cm above a padded surface for a maximum of 60 s. If the animal fell from the grid, the latency from the time of inversion to the time of fall was recorded. For animals that did not fall from the grid, the holding time was recorded as 60 s.

### Loss-of-Righting Reflex Test

In the loss-of-righting reflex test, which provides an indication of profound sedation/anesthesia, mice were placed in a cage following treatment and if they began showing signs of sedation (wobbly ataxic gait and disoriented movements) they were taken out of the cage and placed on a paper stage. Animals were gently turned onto their backs every 10 s. If an animal immediately regained normal posture, it was scored as not impaired whereas an animal that did not immediately right itself was scored as impaired. For impaired animals, onset of the loss-of-righting reflex was the time after treatment administration at which the animal first exhibited impairment. Regain of righting reflex was the time after treatment administration that the animal first regained the ability to right itself when it was able to do so 3 times during successive 10 s periods. The total time for loss-of-righting reflex (“total impaired time”) was calculated as the difference between the time of the onset and regain of righting reflex.

### Brain Pharmacokinetic Analyses

At various times after treatment administration, brains were removed and were separated on ice into 3 sections with a scalpel (olfactory bulb, forebrain with midbrain, cerebellum with attached pons, and medulla) and fast-frozen on dry ice. Cross contamination was eliminated by cleaning the scalpel between cuts. A separate scalpel was used for each brain. Brain samples were processed with a modified approach. Each separated brain section was placed in a 2 ml cryovial prefilled with 3 mm zirconium beads (SPEXSamplePrep, Metuchen, NJ). One milliliter, 0.5 ml, or 0.25 ml of H_2_O was then added to the forebrain/midbrain, cerebellum, or olfactory bulb samples in each vial, respectively. The vials were shaken with a Geno/Grinder® Automated Tissue Homogenizer and Cell Lyser (SPEXSamplePrep, Metuchen, NJ) at 1750 strokes per min for 2 min. Two hundred microliters of the resulting homogenate was transferred to a fresh microcentrifuge tube. One hundred microliters of 100 ng/ml D4-allopregnanolone solution in acetonitrile was then added to the tube. After a brief vortex, the tube was centrifuged at 17,000×*g* for 5 min. The supernatant was collected for solid phase extraction (SPE).

To prepare a brain homogenate calibration curve, blank whole brains were obtained from untreated mice and homogenized with 1 ml of H_2_O using the same homogenization method described above. The homogenates obtained from 10 brains were pooled together and mixed well. One hundred and ninety-seven microliters of the blank brain homogenate was dispensed into fresh microcentrifuge tubes and 2 µl of standard allopregnanolone stock solution in methanol at various concentration was spiked into the brain homogenate in each tube to make calibrators at the following concentrations: 0, 2.5, 5, 15, 50, 100, 250, 500, 1000, 1500, 3000, and 4500 ng/ml. Six quality control (QC) samples were also prepared for intra-batch accuracy assessment at the following concentrations: 2.5, 7.5, 500, and 900 ng/ml. One hundred microliters of 100 ng/ml D4-allopregnanolone solution in acetonitrile was then added to the tube. After a brief vortex, the tube was centrifuged at 17,000×*g* for 5 min. The supernatant was collected for SPE.

Two hundred-microliter aliquots of the resulting brain homogenate supernatant from calibrators, QC samples, or mouse brain samples were loaded onto and passed through a Waters Oasis HLB 1 ml (30 mg reversed-phase sorbent) SPE cartridge that had been pre-equilibrated by passage of 1 ml acetonitrile and then 1 ml of H_2_O. The SPE cartridge was then washed once with 1 ml of 40%/60% (v/v) acetonitrile/H_2_O, and the analytes were eluted with 3 ml of 100% acetonitrile. The eluents were dried by air stream and reconstituted with 200 μl of 50%/50% (v/v) acetonitrile/H_2_O. Ten-microliter aliquots of the solution were injected into the LC-MS/MS for quantification as described in Zolkowska et al. [63]. The lower limit of quantification in plasma is 2.5 ng/ml. The accuracy at each QC concentration was 13.78%, 15.31%, 2.49%, and 4.17% deviation, respectively; the precision (%CV) was 29.64%, 8.33%, 1.99%, and 4.43%, respectively.

### Data Analysis

The 95% confidence interval (CI) values of ratios were calculated using the Fieller method with an application provided by GraphPad Software (https://www.graphpad.com/quickcalcs/). *t* test and chi-square analyses were performed with GraphPad Prism (GraphPad Software, San Diego, CA). Brain pharmacokinetic parameters were derived using Phoenix WinNonlin (Certara, Princeton, NJ).

## Results

### Comparison of Intranasal Allopregnanolone, Midazolam, and Diazepam on Seizure Threshold

**Fig. 1:**
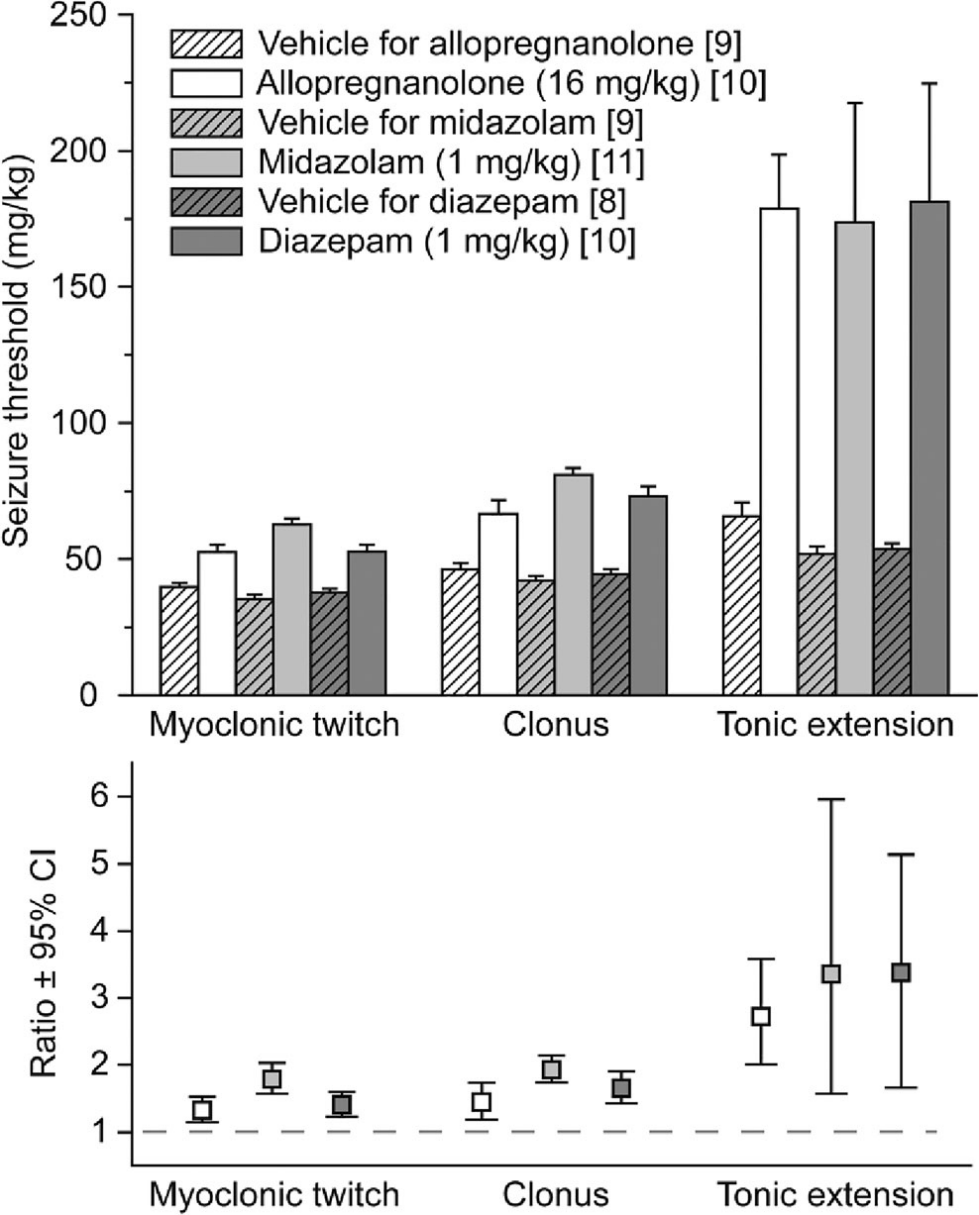
Effects of intranasal allopregnanolone, midazolam, and diazepam on thresholds for myoclonic twitch, clonus, and tonic extension in the timed intravenous PTZ seizure threshold test in mice. The PTZ infusion was initiated 10 min after the completion of the intranasal administration. In the upper panel, bars represent the mean ± S.E.M of the seizure threshold values; numbers of animals are indicated in brackets. The lower panel shows the ratio of the seizure threshold with drug treatment to the seizure threshold of the corresponding vehicle with 95% confidence intervals (CIs). Symbol fills are the same as in the upper panel. All ratio values are greater than 1 (dashed line) inasmuch as all 3 drugs increased the threshold to all 3 seizure signs. For each seizure sign, the 95% CIs of the 3 drugs overlap indicating that the ratios for each of the seizure signs for the 3 drugs are similar. The 95% CI values of the ratios were calculated using the Fieller method

The effects of intranasal allopregnanolone at a dose of 16 mg/kg on the thresholds for myoclonic body twitch, clonus, and tonic extension were assessed in the timed intravenous PTZ threshold test. As shown in Fig. 1, the thresholds for all seizure endpoints were increased compared to vehicle 10 min following the completion of nasal administration. The extent of threshold elevation was similar for the myoclonic twitch and clonus endpoints and higher for the tonic extension endpoint (ratio of mean threshold values in allopregnanolone and vehicle groups, 1.3, 1.4, and 2.7, respectively). For comparison, we also studied midazolam and diazepam. At a dose of 1 mg/kg, the two benzodiazepines produced comparable elevations in the thresholds to the three seizure endpoints.

### Dose–Response Relationship for Elevation of PTZ Seizure Threshold by Intranasal Allopregnanolone

**Fig. 2:**
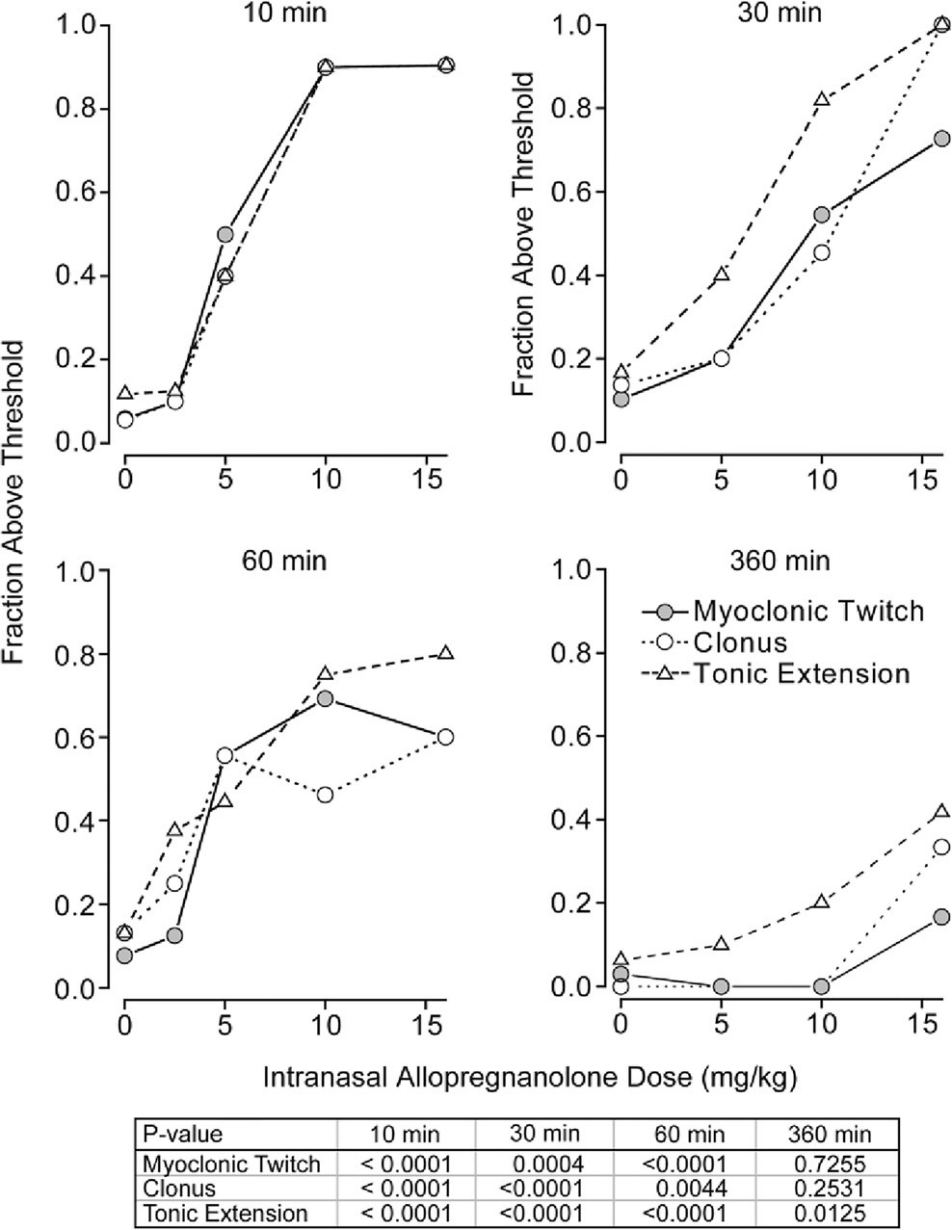
Dose–response relationships for elevation of the myoclonic twitch, clonus, and tonic extension thresholds by allopregnanolone in the timed intravenous PTZ seizure threshold test at various times after dosing. Doses of allopregnanolone tested were 2.5 mg/kg, 5 mg/kg, 10 mg/kg, and 16 mg/kg. The PTZ infusion was initiated at 10 min, 30 min, 60 min, and 360 min after completion of intranasal administration. With each dose group, a contemporaneous group of vehicle-treated animals was tested. A cutoff value was set near the upper limit of the threshold values in the contemporaneous vehicle groups (35–49 mg/kg, 41–53 mg/kg, and 55–69 mg/kg for myoclonic twitch, clonus, and tonic extension, respectively). The fraction of animals with threshold values above the cutoff value is plotted; values for the vehicle groups were averaged and plotted at 0 mg/kg. Each group consists of 7 to 21 animals. ED_50_ values for each seizure endpoint in the 10-min group, determined by straight-line fit of the log-transformed values, were, respectively, 5.4 mg/kg, 5.7 mg/kg and 5.6 mg/kg for myoclonic twitch, clonus, and tonic extension. *p* values determined by chi-square analysis are shown in the table

The efficacy of different intranasal allopregnanolone doses for elevation of the 3 seizure sign thresholds in the timed intravenous PTZ seizure threshold test was determined at 10 min, 30, 60 min and 360 min after completion of dosing. As shown in Fig. 2, a statistically significant (*p* < 0.05) dose-dependent relationship was obtained at each time point except for myoclonic twitch and clonus at 360 min. The mean ED_50_ value determined as described in the caption to Fig. 2 is 5.6 mg/kg. To assess the potency of allopregnanolone when administered by a more conventional parenteral route of administration, we determined the threshold values 10 min following intramuscular delivery of allopregnanolone at a dose of 0.5 mg/kg in 10 animals. The fraction above threshold values were 0.8, 0.8, and 0.9 for myoclonic twitch, clonus, and tonic extension, respectively. This compares to values in 10 contemporaneous vehicle-treated animals of 0.1, 0.1, 0 (*p* < 0.0001). The fraction above threshold values correspond in magnitude by interpolation to a 10.3 mg/kg intranasal dose.

### Time Course of Intranasal Allopregnanolone Effects on Tonic Extension Endpoint in the PTZ Seizure Threshold Test

**Fig. 3:**
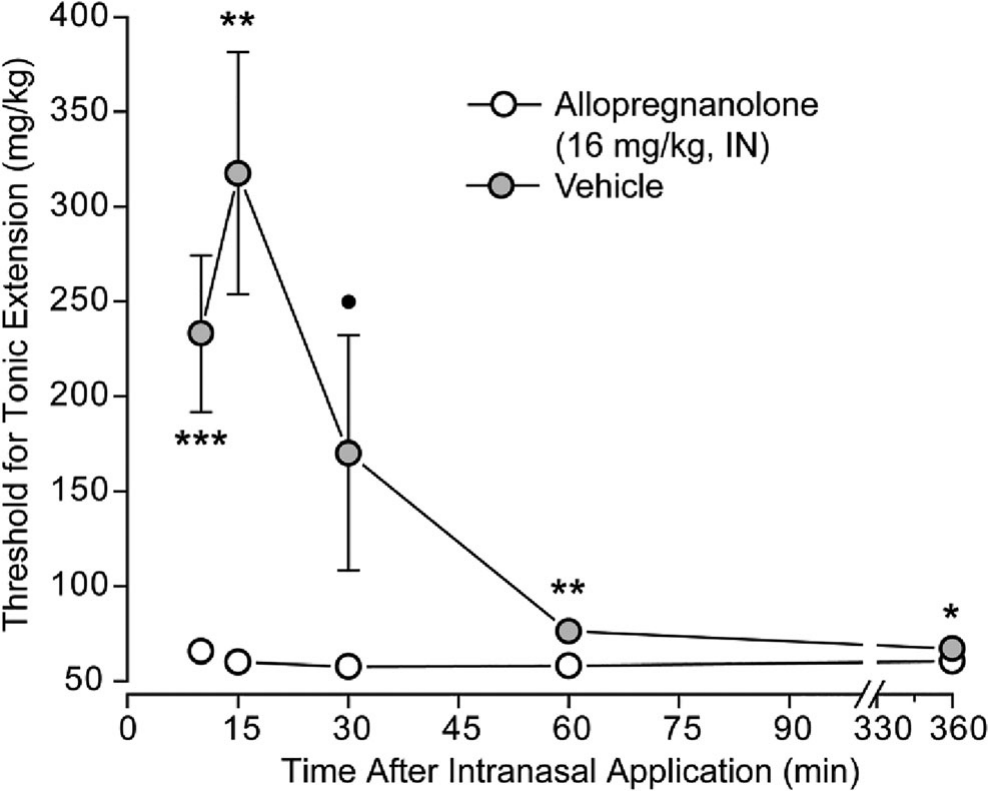
Time course for the effect of intranasal allopregnanolone (16 mg/kg) and vehicle on tonic extension threshold in the timed intravenous PTZ seizure threshold test in mice. Data points indicate the mean ± S.E.M of the seizure threshold values in 11–19 animals. Error bars smaller than the size of the symbols are not shown. *, *p* < 0.05; **, *p* < 0.01, ***, *p* < 0.001; ●, *p* = 0.082

To obtain a more complete assessment of the time course, groups of animals were evaluated in the timed intravenous PTZ seizure threshold test with intervals between intranasal dosing of vehicle and allopregnanolone (16 mg/kg) of 10, 15, 30, 60, and 360 min. As shown in Fig. 3a, a statistically significant increase in threshold was obtained at the earliest (10 min) allopregnanolone group compared with the corresponding vehicle group. The maximum elevation occurred at 15 min (ratio of mean thresholds in allopregnanolone and vehicle groups, 5.3; 95% CI: 3.1–7.7). Consistent with the results of the experiment in Fig. 2, a smaller elevation was obtained at 60 min (ratio, 1.3; 95% CI: 1.1–1.5). At 360 min, there continued to be a statistically significant elevation in threshold but the magnitude was small (ratio, 1.1; 95% CI: 1.0–1.2).

### Effects of Intranasal Allopregnanolone in the Maximal PTZ Seizure Test

As an alternative to the PTZ threshold test, we assessed the effect of pretreatment with intranasal allopregnanolone in the maximal PTZ seizure test. All 30 vehicle-treated animals receiving a maximal dose of PTZ exhibited a sequence of myoclonic twitch, clonus, and tonic extension with the following mean ± S.E.M. latencies: 63.9 ± 1.7 s, 76.2 ± 2.5 s, and 204.1 ± 17.1 s. Intranasal allopregnanolone (10 mg/kg) treatment was administered over a 3.5 min period and PTZ was administered 5 min after dosing. The pretreatment increased the latencies to the onset of the 3 seizure signs. The ratios of the latencies in the allopregnanolone group to the vehicle group were as follows: myoclonic twitch, 1.6 (95% CI: 1.2–2.0); clonus, 1.9 (95% CI: 1.2–2.6); tonic extension, 4.0 (95% CI: 2.3–5.8). All of the allopregnanolone-treated animals exhibited myoclonic twitch and clonus. However, of the 18 animals tested, 5 failed to exhibit tonic extension; the latency was taken to be the entire 30 min (1800 s) observation period. In a similar experiment, the interval between completion of intranasal dosing to PTZ administration was 10 min. The ratios in this experiment were as follows: myoclonic twitch, 1.3 (95% CI: 1.2–1.5); clonus, 1.5 (95% CI: 1.2–1.8); tonic extension, 4.4 (95% CI: 2.4–6.6). As in the prior experiment, allopregnanolone-treated animals exhibited myoclonic twitch and clonus but of the 14 animals tested, 5 failed to exhibit tonic extension.

### Effects of Intranasal Allopregnanolone in the Intravenous Picrotoxin and Bicuculline Threshold Tests

**Fig. 4:**
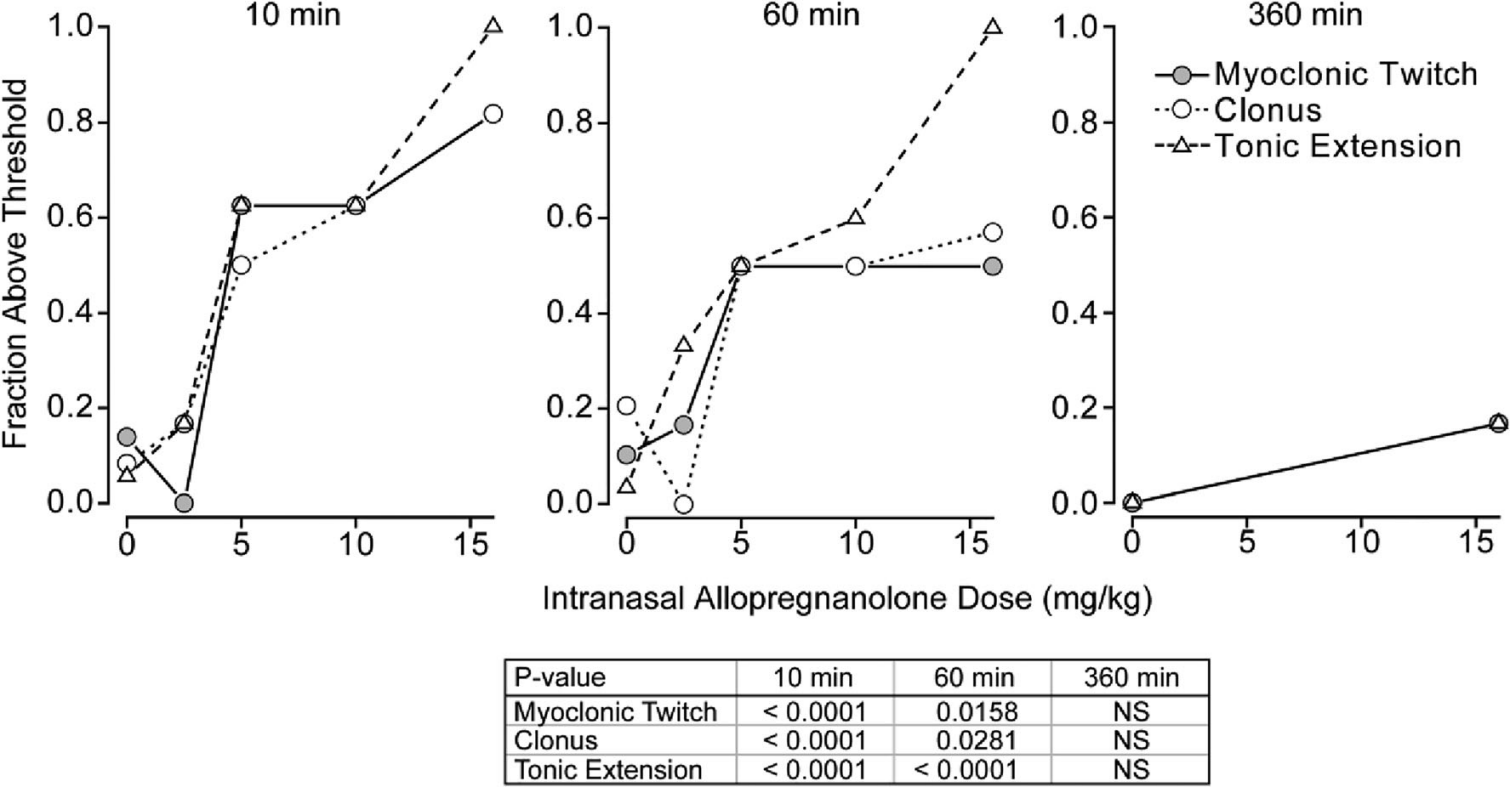
Dose–response relationships for elevation of the myoclonic twitch, clonus, and tonic extension thresholds by allopregnanolone in the timed intravenous picrotoxin seizure threshold test at various times after dosing. Doses of allopregnanolone tested were 2.5 mg/kg, 5 mg/kg, 10 mg/kg, and 16 mg/kg, except in the case of the 360-min group where only 16 mg/kg was tested. The picrotoxin infusion was initiated at 10 min, 60 min, and 360 min after completion of intranasal administration. With each dose group, a contemporaneous group of vehicle-treated animals was tested. A cutoff value was set near the upper limit of the threshold values in the contemporaneous vehicle groups (16.1–17.0 mg/kg, 21.0–22.3 mg/kg, and 27.0–29.0 mg/kg for myoclonic twitch, clonus, and tonic extension, respectively). The fraction of animals with threshold values above the cutoff value is plotted; values for the 3 vehicle groups were averaged and plotted at 0 mg/kg. Each group consists of 6 to 16 animals. ED_50_ values for each seizure endpoint in the 10-min group, determined by straight-line fit of the log-transformed values, were, respectively, 6.4 mg/kg, 6.2 mg/kg, and 5.1 mg/kg for myoclonic twitch, clonus, and tonic extension. *p* values determined by chi-square analysis are shown in the table

**Fig. 5:**
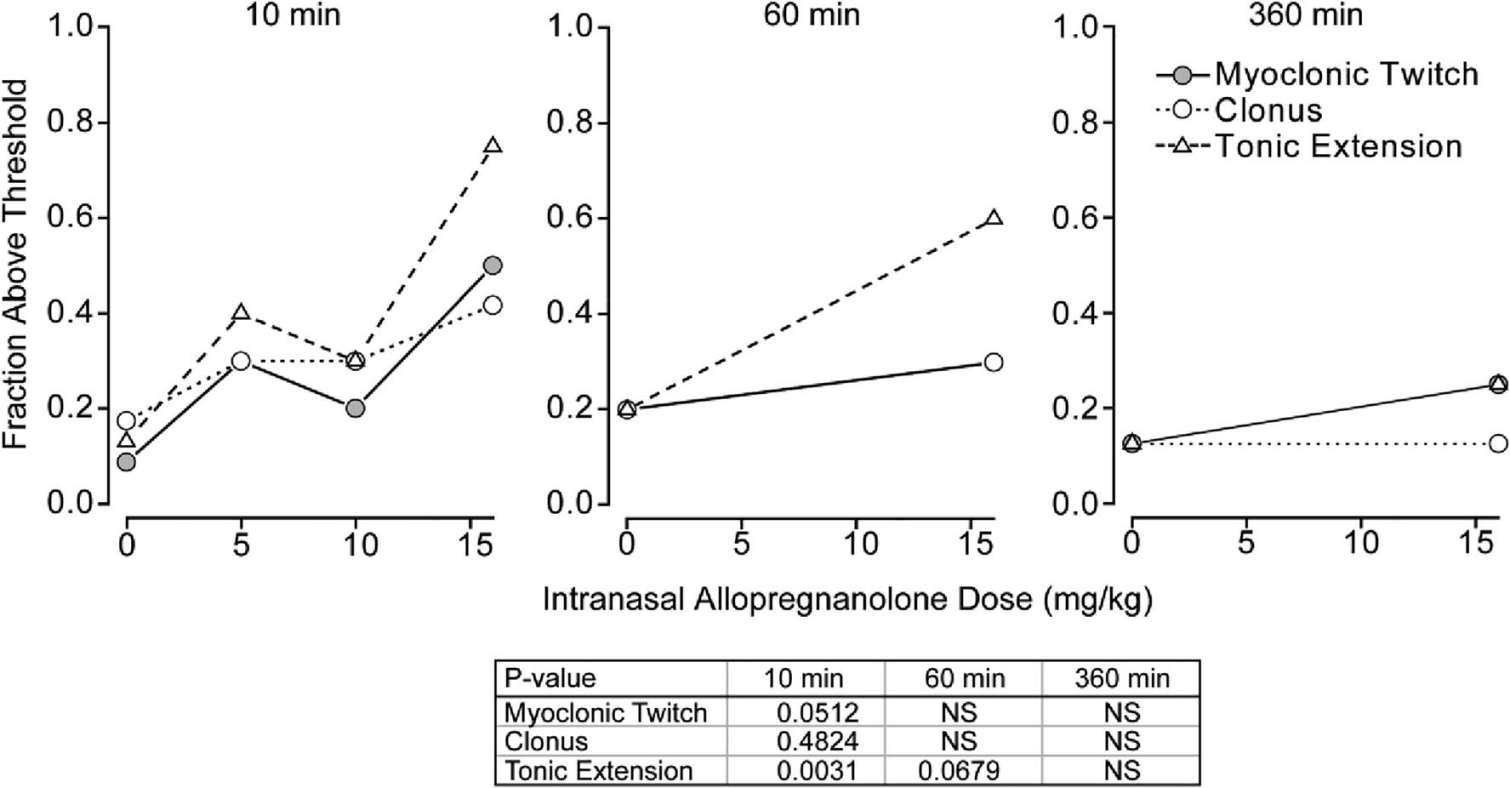
Dose–response relationships for elevation of the myoclonic twitch, clonus, and tonic extension thresholds by allopregnanolone in the timed intravenous bicuculline seizure threshold test at various times after dosing. Doses of allopregnanolone tested were 5 mg/kg, 10 mg/kg, and 16 mg/kg. The bicuculline infusion was initiated at 10 min, 60 min, and 360 min after completion of intranasal administration. With each dose group, a contemporaneous group of vehicle-treated animals was tested. A cutoff value was set near the upper limit of the threshold values in the contemporaneous vehicle groups (0.578–0.740 mg/kg, 0.659–0.810 mg/kg, and 0.924–1.110 mg/kg for myoclonic twitch, clonus, and tonic extension, respectively). The fraction of animals with threshold values above the cutoff value is plotted; values for the 3 vehicle groups were averaged and plotted at 0 mg/kg. Each group consists of 8 to 14 animals. The ED50 value for tonic extension in the 10-min group, determined by straight-line fit of the log-transformed values, was 9.9 mg/kg. *p* values determined by chi-square analysis are shown in the table

The efficacy of different intranasal allopregnanolone doses for elevation of the 3 seizure sign thresholds in the timed intravenous picrotoxin and bicuculline seizure threshold test was determined at 10 min, 60 min and 360 min after completion of dosing. As shown in Fig. 4, a statistically significant (*p* < 0.05) dose-dependent relationship was obtained at the 10-min and 60-min time points but not at the 360-min time point. The mean ED_50_ value determined as described in the caption to Fig. 4 is 5.9 mg/kg. A similar experiment was conducted with intravenous bicuculline (Fig. 5). A significant elevation of seizure threshold for the tonic extension endpoint only was observed at 10 min but not at 60 min or 360 min after intranasal allopregnanolone. Based on this endpoint only, the ED_50_ value at 10 min was estimated as 9.9 mg/kg, indicating that allopregnanolone may be less potent in the bicuculline model than in the PTZ or picrotoxin models.

### Comparison of Intranasal Allopregnanolone, Midazolam, and Diazepam on Motor Impairment

**Fig. 6:**
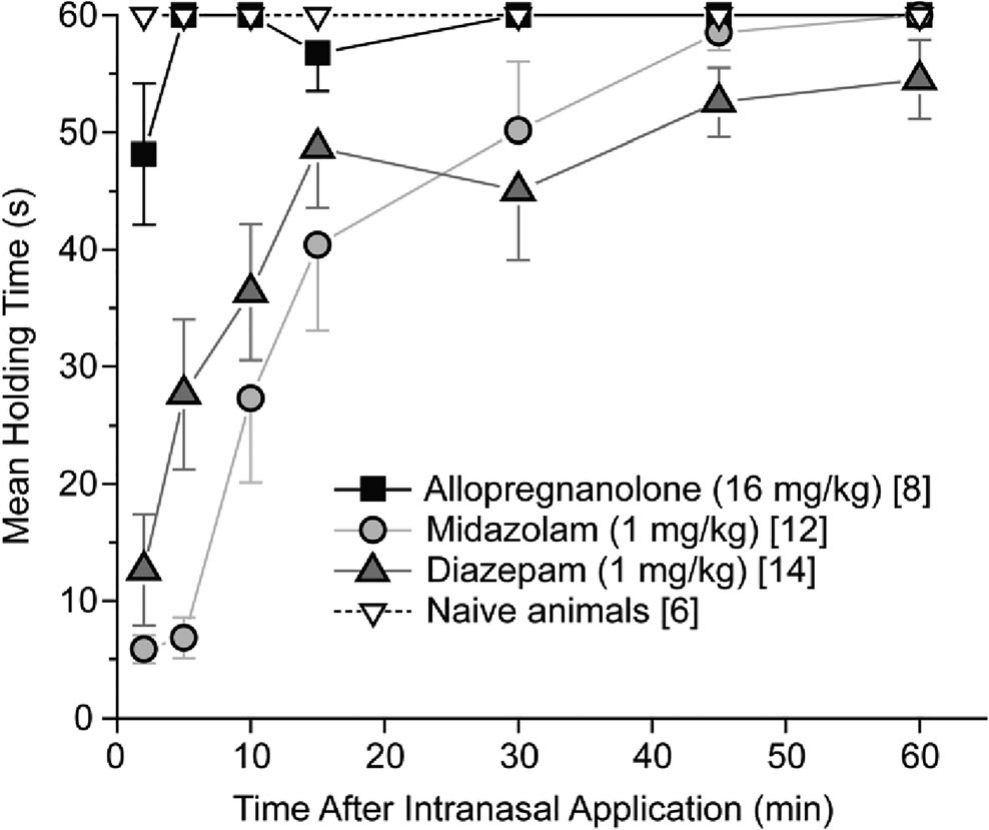
Propensity for intranasal allopregnanolone, midazolam, and diazepam to cause motor impairment in mice as assessed with the horizontal screen test. For comparison, naive mice that did not receive treatment were tested. Observations were made repeatedly in each mouse at the times indicated after the completion of the intranasal administration. Data points represent the mean ± S.E.M. of the holding times for each animal; numbers of animals are indicated in brackets. Drug-treated animals were used only once. Area under the curve (AUC) values for each animal were calculated by the trapezoid rule. Statistical comparisons were made with a two-tailed, two-sample independent sample *t* test: allopregnanolone vs. naive animals, *p* = 0.98; midazolam vs. diazepam, *p* = 0.98; allopregnanolone vs. midazolam, *p* = 0.00062; allopregnanolone vs. diazepam, *p* = 0.00030

The horizontal screen test was used to assess motor impairment following intranasal administration of the test substances (Fig. 6). Naive animals that did not receive a test substance did not fall from the grid during the 60 s observation period. Similarly, most (5 of 8) animals receiving intranasal allopregnanolone (16 mg/kg) did not fall from the grid; the remainder fell only at the 2-min time point (after 28 s and 38 s) and 1 at the 15-min time point (after 37 s). By contrast all midazolam- and diazepam-treated animals fell from the grid. The mean hold times were 5.8 ± 1.2 s and 12.6 ± 4.8 s, for midazolam and diazepam, respectively, at the 2-min time points. Comparisons of the groups were made by calculating the area under the mean holding time versus time after nasal administration curves for each animal. As noted in the caption to Fig. 6, the curves for untreated (naive) animals and allopregnanolone were not significantly different by this measure. Similarly, the curves for the midazolam and diazepam groups were not significantly different. In contrast, comparisons between the allopregnanolone and midazolam, and allopregnanolone and diazepam groups were significantly different.

**Fig. 7:**
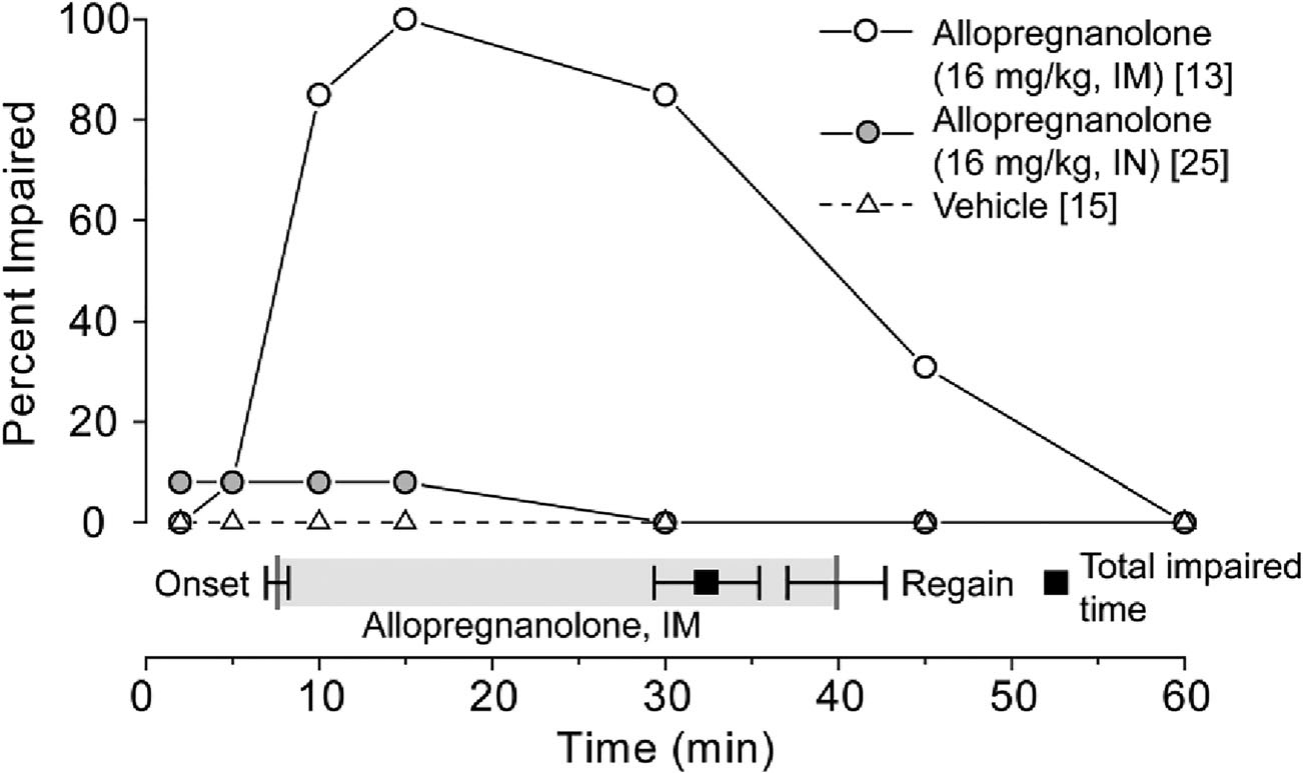
Comparison of the propensity of intranasal (IN) and intramuscular (IM) allopregnanolone to cause loss-of-righting reflex. Each data point in the line graph indicates the percentage of total animals in the treatment group at each time point exhibiting impairment. The total number of animals in each treatment group is indicated in the caption in brackets. The bar below the line graph indicates the mean ± S.E.M. times for onset and recovery of loss-of-righting reflex and the total impaired time in the group receiving IM allopregnanolone. A similar analysis for IN allopregnanolone was not made since only 2 of 25 animals experienced loss-of-righting reflex

Another means of assessing the propensity of intranasal allopregnanolone to cause neurological impairment (motor impairment, sedation, or general anesthesia) is with the loss-of-righting reflex test. As shown in Fig. 7, 16 mg/kg intranasal allopregnanolone had low propensity to impair neurological function significantly as only 2 of 25 animals exhibited loss-of-righting reflex during a 60 min observation period after dosing. By contrast, all of the 10 animals receiving the same dose of allopregnanolone intramuscularly exhibited loss-of-righting reflex.

### Brain Allopregnanolone Levels Following Intranasal Delivery

**Fig. 8:**
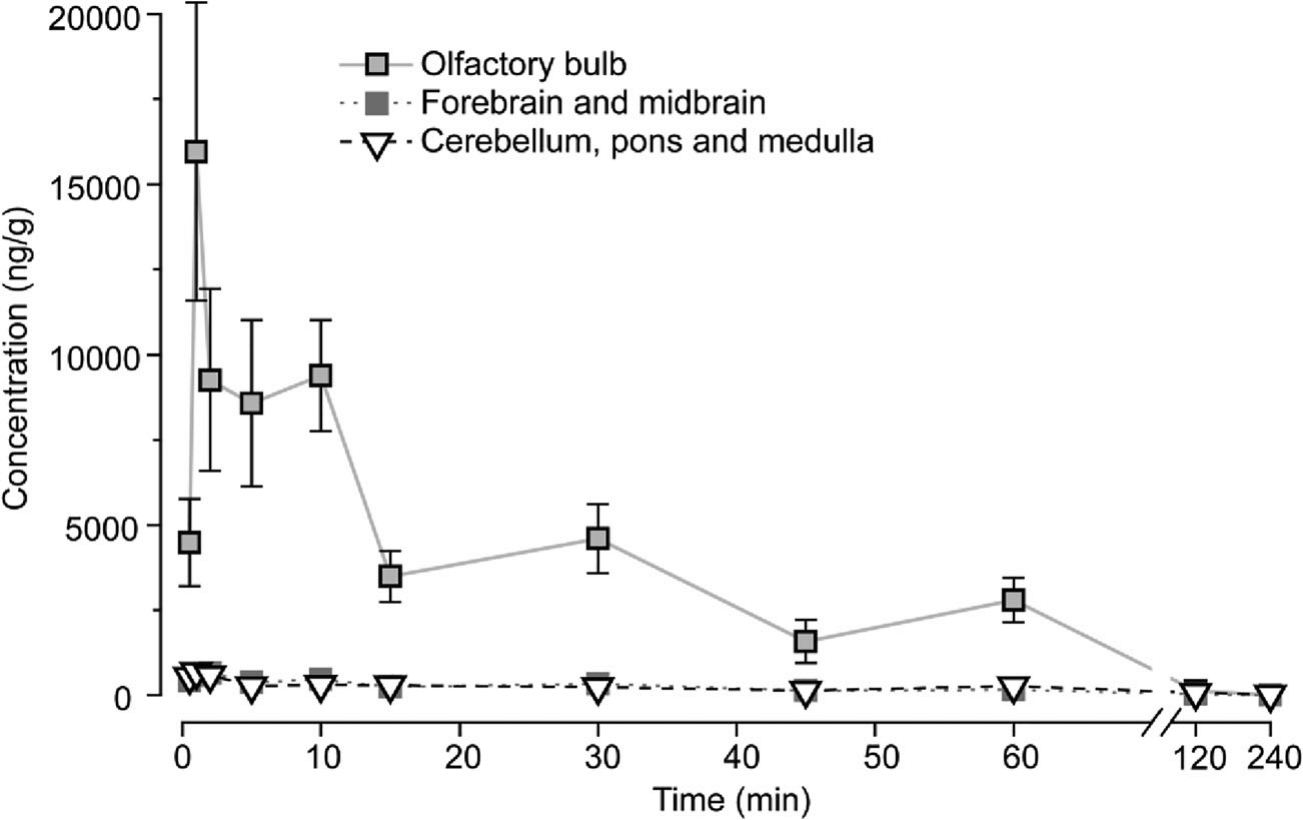
Mean ± S.E.M. brain region concentrations of allopregnanolone following intranasal administration. The allopregnanolone dose was 10 mg/kg. Tissue was collected at various times after the completion of the intranasal administration. Each time point represents a separate group of 8–10 mice

Figure 8 and Table 1 show the results of measurements of allopregnanolone in various brain regions following intranasal dosing with 10 mg/kg allopregnanolone. Olfactory bulb concentrations are very high in relation to the forebrain and midbrain, and cerebellum and brain stem. The olfactory bulb *C*_max_ value is 24-fold that of the other brain regions. The *T*_max_ values of 1–2 min indicate rapid uptake and transport into the brain and are consistent with the rapid antiseizure activity observed with intranasal delivery. The nominal *T*_max_ values reported were determined from time measurements made at the end of the 3.5 min slow instillation of allopregnanolone into the nose. Therefore, the true *C*_max_ is not known with precision but is likely <5 min.

**Table 1:** Pharmacokinetic parameters derived from the data presented in Fig. 8. CB & BS = cerebellum, pons and medulla; FB & MB = forebrain and midbrain; OB = olfactory bulb. AUC = area under the curve. *T*_max_ values are based on time after completion of 3.5 min intranasal administration

Tissue	*C*_max_ (ng/g)	*T*_max_ (min)	*T*_½ (2~120 min)_ (min)	*AUC*_0–120 min_ (min·ng/g)	*AUC*_0–∞_ (min·ng/g)
CB & BS	672	1	49.0	24,307	28,861
FB & MB	648	2	24.5	21,128	22,229
OB	15,960	1	19.6	349,718	352,934

## Discussion

Previous reports have indicated that intranasally administered benzodiazepines enter the brain via absorption into the systemic circulation followed by transport across the blood–brain barrier [31]. Thus, in a study with rabbits and rats, intranasal administration of diazepam in a microemulsion formulation produced a homogenous drug distribution in the brain with concentrations in the olfactory bulb no greater than those in other brain regions or the brain as a whole [32]. In a separate study in rats, the relative exposure of midazolam in the olfactory bulb and olfactory tract following intranasal deposition of an aqueous suspension of midazolam was no different from the relative exposure in the rest of the brain, and the ratio of exposures following intranasal and intravenous delivery in the olfactory areas did not substantially exceed the ratio in the rest of the brain [28]. These results indicate minimal brain-to-nose delivery of the highly lipophilic benzodiazepines; the bulk of distribution to the brain is via the venous circulation. In striking contrast, we found that allopregnanolone exposures in the olfactory bulb were 12-fold to 16-fold higher than those in the extra-olfactory bulb brain. The inhomogeneous distribution pattern provides strong evidence of nose-to brain delivery, as was originally demonstrated for diverse biomolecules [17, 58] including the steroids progestesterone, pregnenolone [16], and testosterone [5].

The speed of nose-to-brain delivery of allopregnanolone is striking (<30 s). Rapid nose-to-brain delivery has been previously observed for the other steroids [16]. Nose-to-brain delivery is believed to occur along olfactory nerve projections to the olfactory bulb and trigeminal nerve projections to the brainstem [19, 37]. Transport across the nasal olfactory or respiratory epithelium is followed by entry into perineural, perivascular, and lymphatic compartments. The surface of the olfactory epithelium consists of sustentacular (supporting) cells and exposed olfactory sensory receptor neurons. Movement across the epithelium can occur by transcellular or paracellular routes. Transcellular transport represents a combination of diffusion and transporter-mediated uptake and efflux [18, 34]. Tight junction proteins restrict paracellular movement but a lipophilic small molecule such as allopregnanolone is susceptible to transcellular transit. Following transfer across the olfactory epithelium, the allopregnanolone must then travel rapidly through the cribiform plate to the olfactory bulb. The high velocity of delivery is not consistent with rates of intracellular axonal transport or diffusion [38]. Rather, following epithelial transit, it has been proposed that rapid delivery in the brain may occur via convective transport in perivascular spaces driven by arterial pulsations [22, 36]. It is interesting to speculate that there could be a structurally specific transport mechanisms, perhaps at the level of the epithelium, that admit certain steroids but not other small lipophilic molecules such as the benzodiazepines diazepam and midazolam. This hypothetical transport system may play a physiological role in the signaling function of steroids inasmuch as there are receptors for sex steroids including progesterone in the olfactory bulb [42] and all olfactory bulb neurons possess GABA_A_ receptors [44], as is the case for neurons in brain regions to which nose-to-brain-delivered allopregnanolone may be subsequently transported. It is noteworthy that other steroids including antiinflammatory steroids are commonly administered intranasally, such as for the treatment of allergic rhinitis. Such steroids are intended to act locally and commonly used agents such as fluticasone propionate have negligible systemic availability, although older agents such as flunisolide exhibit substantial systemic absorption [39]. It has been speculated that these steroids are transported into the brain but experimental support is lacking [35].

Allopregnanolone is well recognized to have antiseizure properties, with potent activity against seizures induced by GABA_A_ receptor antagonists such as PTZ, picrotoxin, and bicuculline [6, 33, 47, 51]. Intranasal delivery of allopregnanolone raised the PTZ, picrotoxin, and bicuculline seizure thresholds and prolonged the latency to onset of seizures in the maximal PTZ seizure test. The onset of action was rapid. An effect on seizure threshold was evident the first time it was tested (10 min, which is 13.5 min after nasal delivery was initiated). The elevation of PTZ seizure threshold occurred in a dose-dependent fashion, with ED_50_ of 5.6 mg/kg in the PTZ threshold test and 5.9 mg/kg in the picrotoxin threshold test. An unexpected finding in this study was that intranasal allopregnanolone at the dose tested caused minimal motor impairment or loss-of-righting reflex, whereas doses of diazepam and midazolam that produced a similar elevation in seizure threshold were associated with profound motor impairment. We speculate that the dissociation occurs because allopregnanolone administered intranasally directly accesses brain structures relevant to seizures avoiding brain regions that are associated with motor impairment whereas the benzodiazepines, which enter the brain exclusively through the vasculature, are distributed evenly throughout the brain, not only inducing antiseizure effects but also influencing regions subserving motor function and level of consciousness. The relevant brain regions for PTZ seizure protection in rodents including mice, are the hippocampus (especially the dentate gyrus), the amygdala, and the piriform cortex [2, 3, 7, 15, 61]. It is therefore of interest that olfactory bulb axons project directly to these same regions [24, 29], so that there is a potential physical pathway from the nose to the relevant brain regions. Indeed, following intranasal administration of progesterone, high levels were rapidly recovered (< 2 min) not only in the olfactory bulb but also hippocampus [16]. Sedation and motor impairment caused by GABA_A_ receptor–positive modulators are believed to result from actions of the drugs on brainstem structures such as the pontine reticular formation and the dorsal raphé nucleus as well as the hypothalamus [59]. Effects on other regions of the hindbrain including the cerebellum may also mediate the motor side effects of these agents. As demonstrated by the results of Fig. 8, the fraction of the administered dose arriving in these brain regions following intranasal delivery is minimal. By circumventing delivery to these structures, intranasal allopregnanolone may avoid adverse effects but still permit a therapeutic action, at least for the treatment of seizures and epilepsy and possibly for other clinical uses.

Our results demonstrate that intranasal delivery is a feasible route of administration for allopregnanolone and can overcome the poor oral bioavailability of the neuroactive steroid. To the extent that poor oral bioavailability of allopregnanolone is due to first-pass hepatic metabolism, this is avoided by nasal absorption [1]. However, it is recognized that with nasal delivery, a large percentage of the dose may still be transported into the gastrointestinal tract by mucociliary clearance. Ingested allopregnanolone would have only minimal bioactivity but would reduce the bioavailability following intranasal administration. Indeed, while we found intranasal allopregnanolone to be effective in diverse seizure tests, the overall potency was low compared to intramuscular dosing (about 20-fold less potent). The potency difference may be due to reduced bioavailability because of swallowing or other loss of the dose. Interestingly, the rate of rise of allopregnanolone in the brain after nasal delivery is even faster than with intramuscular delivery [63], indicating that slow pharmacokinetics is not a factor. Allopregnanolone is highly bound to plasma proteins (> 99%). Although there is extensive entry into the brain with hematogenous delivery [63], protein binding may limit the extent of delivery [45]. Direct nose-to-brain delivery avoids plasma protein binding, which could increase the efficiency of delivery to the brain by this route [5].

The lipophilic nature of allopregnanolone (log P, 4.9) makes it challenging to formulate for parenteral administration. We used the cyclodextrin sulfobutylether-β-cyclodextrin to formulate allopregnanolone for intravenous delivery, in a preparation that was ultimately commercialized [49, 55]. The same excipient was used in the present study to create an aqueous delivery vehicle for intranasal delivery. Cyclodextrins have been used as permeation enhancers for intranasal delivery of various substances [10, 14, 41]. They are believed to enhance permeability across the nasal epithelium by extracting membrane cholesterol, which leads to the opening of tight junctions. Interestingly, cyclodextrins have been found to increase nose-to-brain delivery of a peptide, leading to preferential exposure of certain brain regions, including the olfactory bulb and also the hippocampus in comparison with a cyclodextrin-free formulation [43]. The role the cyclodextrin component of our formulation plays in nose-to brain delivery of allopregnanolone and its regionally selective targeting remains to be investigated. While the nasal toxicity of sulfobutylether-β-cyclodextrin has not been formally investigated, cyclodextrins have been proposed to be safe for installation into the nose based on studies of ciliary beat frequency and the release of intracellular marker compounds [41]. In humans, a sulfobutylether-β-cyclodextrin formulation of midazolam caused mild to moderate transient irritation of nasal and pharyngeal mucosa but no serious side effects [21]. Apart from local effects in the nose which appear to be benign, consideration must be given to the potential for adverse effects if the cyclodextrin excipient is transported into the brain along with the active agent.

In summary, our results for the first time demonstrate that nose-to-brain delivery of the steroid allopregnanolone can have clear functional effects evident with tests of antiseizure activity. We observed that intranasal allopregnanolone fails to cause substantial sedation or motor impairment at antiseizure doses. This is in contrast to the benzodiazepines midazolam and diazepam, which dramatically impair motor function in the horizontal screen test at doses that have comparable antiseizure activity. Our results suggest that intranasal delivery could be a superior route of administration for certain applications, including for the acute treatment of seizures. Intranasal allopregnanolone acts rapidly, within < 5 min and possibly faster. Prior studies with intranasal progesterone in humans also demonstrated rapid absorption (< 2 min) [8]. Although there is clinical evidence that intranasal benzodiazepines terminate seizures rapidly (within < 5 min), pharmacokinetic studies in humans indicate slower absorption with a *T*_max_ of 10–17 min for midazolam [4, 21, 54] and 80–90 min for diazepam [25, 52]. The speed of onset implies that intranasal allopregnanolone could be used to treat acute indications, including acute seizures. In rodents the olfactory epithelium covers a larger area of the nasal mucosa than in humans where olfactory transport of drugs may be less efficient [26]. Although there is experimental evidence suggesting that nose-to-brain delivery can in certain circumstances occur in humans [27, 58], the demonstration of nose-to-brain delivery in rodents does not imply that sufficient brain exposures can be achieved in humans. Studies are required to assess the extent to which the observations reported here are clinically applicable.

## Supplementary information

ESM 1 (PDF 1.19 mb)
